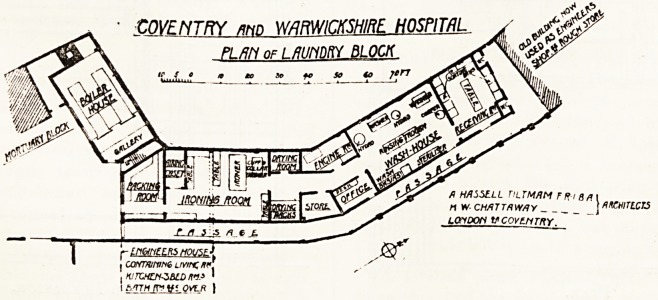# Coventry and Warwickshire Hospital Laundry

**Published:** 1912-11-23

**Authors:** J. A. Rudd

**Affiliations:** Secretary.


### Coventry and Warwickshire Hospital Laundry.

By J. A. RUDD, Secretary.

The new laundry at this hospital occupies a building which was formerly used as a cycle factory called the Round Tower Works, the name being taken from a round tower which stood on the site where the new mortuary has been built. The buildings were adapted and altered for the purpose of a modern institutional laundry; the departments have been so admirably planned that for convenience and economy of working it is scarcely possible to offer suggestions for improvement. The soiled linen arrives at the room for sorting, and from whence it is delivered to the wards, etc., in baskets. The walls of the soiled-linen room, the washhouse, and the engine room have been finished in glazed brick and the floors are in concrete, thus ensuring absolute cleanliness.

Taking the various rooms in the order in which the work proceeds, we have—

1. The soiled linen room, around the walls of which glazed brick sorting bins are built in for the easy dis-

As will be seen from the plan, each department is self-contained. The soiled linen is delivered to the department which has to give it first attention, and is conveyed along the passage at the side, so that no interference with the general work of the other departments through the drawing of trucks to and fro takes place, the soiled work entering at the farthest point and emerging, after it has passed through the various processes, integration of the work before it passes to the washhouse, and in the centre is placed a large table; this room is connected by swing-doors with

2. The washhouse. Here are placed the usual washing machines, hydro extractors, etc., and also a disinfector, in which is placed all foul or infected linen; an important feature of this machine is that the infected linen is put into the machine from the passage outside, thus ensuring that all infected linen is thoroughly disinfected before it comes in contact with the other work in the process of washing.

3. In the passage leading from the washhouse to the ironing room are placed the engine room, laundresses' office, stores, drying room, and drying horses; these are of the usual type and need no special comment.

4. We now reach the ironing room, which is well lit by an overhead light and well ventilated, a Blackman fan being placed in the roof. This room contains a full-sized Decoudin ironer for sheets and large flat work, a shirt and collar ironer, and ironing benches. The hand-irons are heated by gas, and have been found to work extremely well. In this room has been erected a compartment for airing the linen before it passes to

5. The sorting room, where the clean washing for the various departments of the institution is sorted into bins fitted with wooden trays, and where it is finally packed into the baskets ready for dispatch.

The laundry is lit by gas. The question of lighting by electricity was considered by the committee, but, owing to the considerable cost of conveying the mains from the Corporation supply at the time, it was not thought advisable to instal the electric light. The laundry is not yet in telephonic communication with the hospital, but this, as well as all other departments, will soon be connected by a system of inter-communicating telephones. The system of disintegration in the soiled-linen room has made it unnecessary to erect a separate department for the washing of officers' linen which is sometimes to be found in institution laundries, and which must add to the cost of working. Over the sorting and ironing rooms have been placed the engineer's resident quarters. The laundry cost, including building and equipping, £5,340.

### OUR EXPERT'S COMMENTS ON THE COVENTRY AND WARWICKSHIRE HOSPITAL LAUNDRY.

With regard to shape and ventilation the same remarks apply to this plan; but no doubt the architects have been governed by exigencies of site. Here again it seems to us that drying accommodation is, compared with the space given to the washhouse, the ironing room and the receiving room, insufficient, though it may be, of course, ample for the requirements of the hospital. Without knowledge of the amount to be washed, it is impossible to give a definite opinion. Nor are the drying rooms placed so well as in the Chichester Infirmary. It is very convenient to have access to the drying rooms or closets from both washhouse and ironing room.

We hope that the boiler house chimney has some smoke consuming apparatus. Where the laundry is an addition to an old building it is often difficult to place it anywhere else except near the engine house. Wherever possible, however, it should be kept as far away from the boiler house and the chimney as convenience will allow.

Here, again, there is no separation shown on the plan of patients' and staff washing, though there is stated to be a system of disintegration in the soiled-linen room. There seems to be ample space provided for this separation, but by itself ample space is no safeguard; it is essential that there should be a sufficient staff. We know of small hospitals where there are laundries, and where from insufficiency of staff, the patients' and staff washing have to be mixed—a most objectionable practice.

## Figures and Tables

**Figure f1:**